# Aortic Arch Thrombosis Mimicking Interrupted Aortic Arch

**DOI:** 10.1155/2013/948234

**Published:** 2013-04-22

**Authors:** Richard Neal, Katharina Mattishent, Fiona Reynolds

**Affiliations:** ^1^Paediatric Intensive Care, Birmingham Children's Hospital, Birmingham B4 6NH, UK; ^2^University of East Anglia, Medical School, Norwich NR4 7TJ, UK

## Abstract

We report a case of a 32-week-gestation male who presented initially with symptoms suggestive of an interrupted aortic arch. The absence of a VSD prompted further investigations, including a CT angiogram, which revealed that he had an extensive thrombus in his aorta. He underwent a successful aortic thrombectomy with arch repair on cardiopulmonary bypass support. In view of the extensive thrombus, the patient was referred to the haematology team. There was no family history of prothrombotic tendencies.

## 1. Case Report

Baby D was born at 32 weeks of gestation via emergency caesarian section following a period of reduced foetal movements. Antenatal scans were normal, and his mother had a relatively uneventful pregnancy until then. There was however a history of previous term stillbirth (no postmortem) and 2 live preterm births who are otherwise well. His mother was not known to have diabetes or be on any medication. There was no risk for sepsis.

At birth, he was intubated for poor respiratory effort and received surfactant for prematurity. He was also noted to have some hydropic signs with ascites but no pleural or pericardial effusion. His local paediatric team was concerned when his saturations were not improving with worsening metabolic acidosis, poor lower limb pulses, and blood pressure. Prostin was commenced for a possible duct-dependent lesion, and dobutamine was started for inotropic support. At his worst, his blood lactate was 19 mmol/L in his local hospital. He was transferred to Birmingham Children's Hospital for a cardiac opinion.

**Figure 1:**
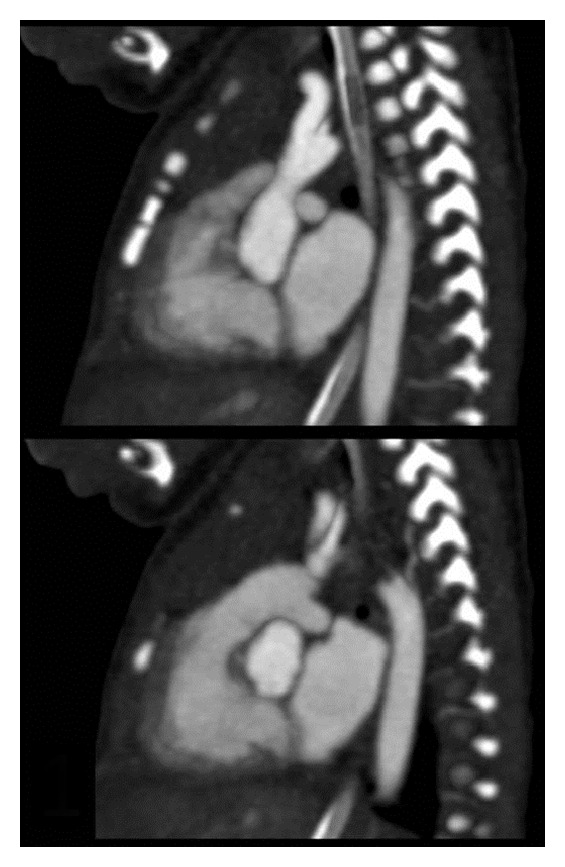
Computed tomography showing the aortic arch which appears to be interrupted with a radiodense lesion distal to the left subclavian artery.

**Figure 2:**
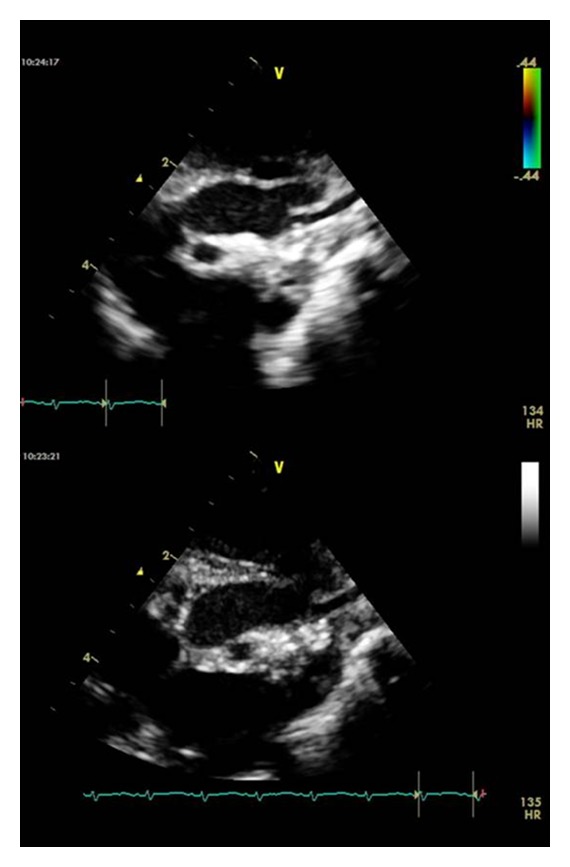
Echocardiogram showing the aortic arch with an echobright lesion within the lumen of the aorta just distal to the left subclavian artery.

In PICU, an initial echo was suspicious of interrupted aortic arch. In view of the absence of a VSD, he had a CT angiogram (Figure 1) that demonstrated an extensive thrombus in his aorta (distal to his left carotid down to his descending aorta). His echo (Figure 2) showed that he had poor left ventricular contractility but no pericardial effusion. Baby D also had a CT head at the same time that revealed extensive intraventricular haemorrhage in lateral, 3rd and 4th ventricles with some subdural bleed. 

He underwent aortic thrombectomy with arch repair on cardiopulmonary bypass support. The surgery was successful, and he had his chest closed 48 hours later. He received adrenaline and milrinone for inotropic support. His echocardiogram continued to show improving cardiac contractility with no left ventricular outflow tract obstruction, and his inotropic support was weaned.

In the initial postoperative period, he developed a transient low urine output. A renal ultrasound at this time showed bilateral, enlarged for gestational age, and echogenic kidneys but no renal tract dilatation. There was poor Doppler signal in both kidneys, but both main renal arteries and veins were patent. 

Heparin infusion of 10 units/kg/hr was given in the postoperative period. The low dose infusion was used in view of his intracranial bleeds. Subsequent to this, he commenced on aspirin. 

In view of the extensive thrombus, Baby D was referred to the haematology team. There was no family history of prothrombotic tendencies. His lupus anticoagulant screen was negative. IgM serology for cytomegalovirus, toxoplasmosis, and rubella was negative.

## 2. Learning Points

A differential diagnosis for interrupted aortic arch is aortic arch thrombosis. In a child with suspected interrupted aortic arch but no VSD, this alternative diagnosis must be more definitively excluded. A CT with contrast of the aortic arch provides additional information regarding the diagnosis of thrombosis. Hypertension in upper limbs is not a usual feature in the neonatal period for interrupted aortic arch and should therefore prompt consideration of alternative diagnoses.

Aortic arch thrombosis is rare in the neonate. Current treatment options include surgical thrombectomy, anticoagulation with low molecular weight heparin (LMWH) or unfractionated heparin, and fibrinolytic treatment. LMWH has been reportedly used with successful resolution of aortic arch thromboses and infra-renal thromboses without any complications [1, 2]. Surgical thrombectomy [3] or thrombolytic therapy [4] have also been reportedly used. Case outcome depends on co-existing or secondary organ dysfunction, with those patients having a severe hypoxic insult being at risk of cerebral ischaemia or infarction with neurological sequelae. 

Evidence to support the appropriate treatment is limited. A literature review was performed by the authors with screening of associated references [5–18]. 18 articles were found with evidence of aortic arch thrombosis. All were case reports or small series. Of those identified, survival ranged widely. Patients with no treatment offered or deemed feasible died, whilst those suitable for treatment with anticoagulation alone appear to have a better prognosis. For the treatment options, survival rates were as follows: surgical thrombectomy 67%, anticoagulation (LMWH or unfractionated heparin) 80%, and thrombolysis 75%. 

Aortic thrombosis is a rare and to date there is no definite treatment option which can be considered as being the “gold standard.” Each case has to be considered individually and all factors taken into account before deciding whether surgical or pharmacological intervention (or a combination of both) is the most appropriate option.
